# Impact of vitamin E in improving comfort, moisture management and mechanical properties of flame-retardant treated cotton fabric

**DOI:** 10.1016/j.heliyon.2023.e23834

**Published:** 2023-12-17

**Authors:** A.T.M. Gulam Moula, Md. Abdullah Al Mamun, Md. Humayun Kabir Khan, Md. Dulal Hosen, Md. Abu Bakar Siddiquee

**Affiliations:** a Department of Textile Engineering, Mawlana Bhashani Science and Technology University, Tangail-1902, Bangladesh; b Department of Textile Engineering, Uttara University, Dhaka-1230, Bangladesh; c Sustainable Advanced Materials Research Institute for Fibre-Biomass and Textiles, Narayanganj-1400, Bangladesh

**Keywords:** Flame retardant, Cotton fabric, Vitamin-E, Comfortability, Crosslinking, Formic acid, Pyrolysis, Moisture management

## Abstract

The aim of this study is to analyze the use of vitamin E to enhance fabric's hand feel, moisture management and fabric strength which are affected due to flame retardant finish. Flame retardant treated fabrics typically become stiff, but the addition of Vitamin E along with emulsifier and binding agent through a vertical padding mangle by two times dipping and nipping results a soft fabric without impairing the flammability properties as determined by 45-degree flammability test. The samples were characterized by FTIR spectra, SEM images, and water contact angle. A new characteristics band is appeared at 1455 cm^−1^ for skeletal vibration of phenyl ring system of alpha tocopherol for Vitamin E measured by Fourier transform infrared spectroscopy (FTIR) that proves the effective attachment of vitamin E with fabric. Hydrophilicity of vitamin E containing sample is discovered after showing 37.629° as a water contact angle at optical tensiometer (Attension theta lite). Additionally, fabric comfort properties, moisture management properties, and mechanical properties were measured by fabric touch tester (FTT), moisture management tester (MMT) machine and tensile strength tester respectively that demonstrate significant affirmative change in almost all indexes of FTT, 43.5 % increase of overall moisture management capacity by MMT and 23 % increase of tear strength by tensile strength tester due to use of vitamin E that effectively compensate lower strength, poor fabric comfort, and low moisture management capability of flame retardant treated fabric.

## Graphical abstract

**Figure:**
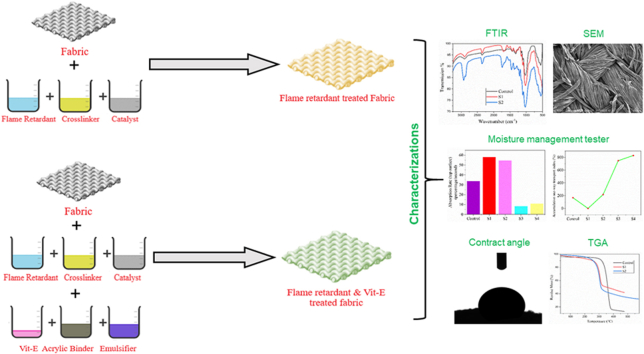

## 1 Introduction

Cotton is a naturally occurring cellulose fiber that is widely used in the textile industry due to its softness, breathability, and absorbency. Cotton is a widely researched crop and researchers from various disciplines have conducted studies on i.e. cotton flammability [1,2] and cotton coloration [[3], [4], [5], [6], [7]] etc. Over the years. However, cotton is also highly flammable due to low ignition temperature, which poses a significant risk in various applications, especially in home textiles such as bedding, curtains, and upholstery [[8], [9], [10]]. When cotton is exposed to a heat source, it undergoes thermal decomposition, releasing flammable gases and forming a charred residue. The flammable gases ignite, causing the cotton to burn rapidly, and the fire can spread quickly to nearby materials. The burning property of cotton can be affected by various factors such as the fiber structure, moisture content, and finishing treatments. To improve the burning property of cotton, flame retardant treatments can be applied. Flame retardants work by either reducing the flammability of the material or by delaying the ignition and spread of the flame. Common flame retardants used in cotton textiles include phosphorous compounds, halogenated compounds, and intumescent agents. The utilization of flame retardant finished fabrics has become increasingly widespread in various fields such as firefighting, military, law enforcement, industrial, high-performance sports, transportation, and even in the creation of sleepwear for children and elderly individuals [[10], [11], [12], [13], [14], [15], [16]]. Achieving flame retardancy (FR) performance in fabric is a crucial concern for fabric engineers. One such approach is utilizing in-built FR fibers like nomex, kevlar, modacrylics, melamine fibers, and polyvinyl chloride, as suggested by Ref. [[2], [17]]. Another method involves incorporating flame retardant additives into the polymer bulk before fiber spinning. Additionally, surface treatments such as impregnation, coating, or spraying with flame retardant compounds, as proposed by Ref. [18,19], can be employed. Generally, a pad-dry-cure approach is used to apply flame retardant agents to the substrate. For instance, N-Methylol-di-methyl-phosphono-propionamide combined with a crosslinking agent such as melamine resin and a catalyst like phosphoric acid has been commonly used for many years as a flame retardant finishing recipe for cotton, as described by Ref. [[20], [21], [22]]. The use of flame retardants in fabrics can increase the fabric's resistance to catch fire, but it can also have adverse effects on the fabric's softness. Flame retardants are chemicals that are added to fabrics to reduce their flammability. These chemicals can alter the structure of the fabric and make it stiffer, which can make it uncomfortable for the wearer. In the case of firefighters, this can be particularly dangerous as it can limit their flexibility and quickness, making it difficult for them to perform their duties effectively in life-threatening situations. The stiffness of flame retardant treated fabrics can also impair heat dissipation, making it difficult for firefighters to regulate their body temperature which results immense sweat formation that might causes massive dehydration in their body [[23], [24], [25]]. In designing protective clothing for firefighters, it is crucial to consider the impact of flame retardants on fabric stiffness and comfort. While previous studies have focused on the mechanical performance of FR-treated fabrics [[9], [26], [27], [28], [29], [30]], there has been limited research on improving the comfort properties of flame retardant treated fabrics or thoroughly characterizing their degree of comfort [[18], [31], [32]]. Softening finish has been explored as an alternative to improve fabric handle, resulting in softer hand, smoother and pliable surface, and better drape. Several studies have investigated the use of softeners on textiles [1,[33], [34], [35], [36]]. Generally speaking, softener-treated textiles have soft hand (supple, pliant, sleek, and fluffy), smooth and pliable surface with more flexibility, and better drape [37]. Although fabric hand and strength are remarkably improved by combined treatment of softener and wetting agent [38] but moisture management properties were not focused during their works. Moreover, wearers of flame retardant fabrics need to face very non-humid environment and they have to stay long time after wearing same garments due to their own nature of profession which causes rapid perspiration results unpleasant feeling until putting off garments [39]. Various researchers have explored highly efficient flame retardants for cotton fabric. For instance Ref. [40], investigated nucleic acids and proteins, which exhibit ignitable properties and other group [41] focused on the development of high-powered flame-retardant cotton fabric. Additionally Ref. [42], developed a bioinspired, water-based, reusable composite with dual uses as a powerful adhesive and flame retardant whereas another group [43] worked on a composite made from bio-waste adorned with phosphorus, which proved highly effective as a flame retardant for cotton fabric. Again group of researcher [44] introduced a polylactic acid (PLA) based intumescent flame retardant solution using cyclodextrin as a carbon source, among other innovative approaches.

No study yet performed to give pleasant feel to the wearers by managing the moisture of the fabric along with maintaining fabric hand feel and strength. Vitamin E is classified as a water-insoluble vitamin and is known chemically as alpha-tocopherol which is frequently employed as an ingredient in functional cosmetics due to its ability to protect the skin from harmful oxidative stress, a leading cause of skin aging, and its excellent moisturizing properties [45]. In this regard, this study is conducted by application of Vitamin E to the flame retardant finishing system without hampering flame retarding property of the fabric along with improving moisture management capacity of the flame retardant fabric which gives pleasant feeling to the consumers. As a result, after certain time wearing of Vitamin E incorporating flame retardant treated garments, users must get some health benefit because vitamin E is skin permeable which results continuous transmission of vitamin E from the fabric to human body due to gentle rubbing of fabrics and human skin. A group of researcher [46] showed that vitamin E is possible to be incorporated with chitosan which bears strong reference for vitamin E to be bonded with cellulose.

Moreover, many traditional textile softeners contain toxic chemicals such as phthalates, formaldehyde, nonylphenol ethoxylates (NPEs), and volatile organic compounds (VOCs). These chemicals can be harmful to human health and the environment. The production of traditional softeners often involves energy-intensive processes and the use of non-renewable resources. This can contribute to a larger carbon footprint and increased environmental degradation. To mitigate these environmental hazards, there has been a growing shift toward the use of eco-friendly or sustainable textile softeners. These alternatives are formulated with biodegradable, non-toxic, and environmentally friendly ingredients to reduce their impact on the environment. One of the key environmental benefits of using vitamin E is its biodegradability. Unlike some synthetic softeners, vitamin E is a naturally occurring compound that can break down more easily in the environment, reducing long-term environmental impact. Preparation of traditional softeners often involve the use of hazardous chemicals that can persist in the environment and pose health risks to workers and ecosystems. Vitamin E offers a safer alternative, reducing the release of harmful substances into the environment.

All the test samples were prepared using the pad-dry-cure technique. Fabric combustibility and absorbency were assessed by flammability test and water contact angle test respectively. Chemical structure of the samples and thermal properties of the fabric were characterized by Fourier-transform infrared (FTIR) spectroscopy and Thermogravimetric analysis (TGA) respectively. Fabric comfort properties were examined by fabric touch tester (FTT) and sweat management properties was evaluated by moisture management tester (MMT). In addition, tear and tensile strength were conducted to test whether such treatment would weaken the fabric.

## 2 Experimental

### 2.1 Materials

The fabric utilized in this study was 100 % cotton, scoured, bleached, and mercerized, with a plain weave pattern. Its specifications can be found in Table 1. The formulations of the six recipes used in this study are listed in Table 2. All the chemicals, except for vitamin E, were provided by Dysin Bangladesh Ltd. Vitamin E was obtained from Drug International, Bangladesh. Table 3 provides detailed information regarding the chemical compounds used in the study.

**Table 1:** Fabric specification.

Mass per unit Area (gm/m^2^)	Thickness (mm)	Ends per inch (EPI)	Picks per inch (PPI)	Yarn count (Ne)	
				Warp	Weft
130	0.287	132	67	40	40

**Table 2:** Formulation of each recipe (in gm/L, by volume).

Chemical compounds utilized in the formulation	Commercial name of the product	Identification number assigned to each sample					
		S1 (gm/L)	S2 (gm/L)				
			S2a	S2b	S2c	S2d	S2e
Flame retardant agent	Pyrovatex CP	300	300	300	300	300	300
Crosslinking agent	SU-268A	30	30	30	30	30	30
Catalyst	Formic Acid	1	1	1	1	1	1
Acrylic Binder	NK Binder	–	10	10	10	10	10
Softening agent	Vitamin E	–	10	20	30	40	50
Emulsifier	Dynol TLD	–	25	40	55	70	90

**Table 3:** A comprehensive explanation of the chemical compounds, including their properties and characteristics.

Product Name	Nature	Properties
Flame retardant agent (Pyrovatex CP)	Organic phosphorus chemical compound.	Finished fabric can hold favorable flame retardancy after 50 times washing.
Crosslinking agent (SU-268A)	Aqueous emulsion of blocked isocyanate.	Excellent performance as adhesion promoter.
Catalyst **(**Formic Acid)	Miscible colorless liquid act as catalyst.	Catalyzes the reaction between flame retardant chemical & cotton.
Acrylic Binder (NK Binder)	Weak anionic milky white emulsion of styrene acrylic copolymer.	Excellent highfastness binder.
Softening agent (Vitamin E)	Compound that is soluble in fat and possesses strong antioxidant and cytoprotective properties.	It can make textiles feel softer and smoother due to the complex glycol sitting in the natural voids of the fabric.
Emulsifier **(**Dynol TLD**)**	Non ionic colorless liquid of easy solubility in water.	Contains good emulsifying properties.

### 2.2 Flame retardant treatment and combination of flame retardant & vitamin-E treatment

**Fig. 1:**
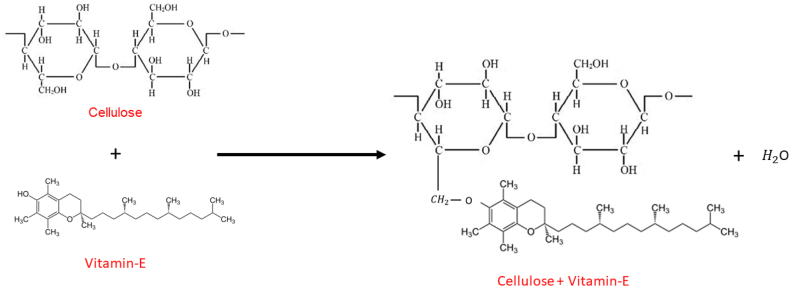
Chemical reaction of vitamin E attachment with cellulose.

The fabrics in this study underwent a conventional process of desizing, scouring, bleaching and mercerization prior to treatment with individual flame-retardant finish and a combination of flame retardant and vitamin E finish. Initially, to prepare a flame retardant padded liquor, the flame-retardant chemical is gently stirred into 100 ml water using a magnetic stirrer. Following the complete dissolution of the flame retardant, the appropriate amount of crosslinking agent is precisely introduced into the solution. Subsequently, formic acid is introduced into the solution. To attain a final volume of 250 ml for the padded liquor, the remaining quantity of water is gradually added while continuously stirring. Secondly, to prepare a vitamin E incorporated flame-retardant padded liquor, Vitamin E is gently stirred into the water using a glass rod, along with the emulsifier. This step ensures the proper dispersion and emulsification of Vitamin E within the aqueous solution. Following the complete dispersion of Vitamin E, the specified quantity of flame retardant is meticulously introduced into the solution. Subsequently, the acrylic binder is added to the solution. Next, the designated crosslinking agent is introduced into the solution. Formic acid, as a catalyst, is then added to the solution. To attain the desired 250 ml total volume for the padded liquor, the additional quantity of water is systematically introduced while maintaining continuous stirring. The proposed chemical reaction of vitamin E attachment with cellulose is appended below in Fig. 1.

**Fig. 2:**
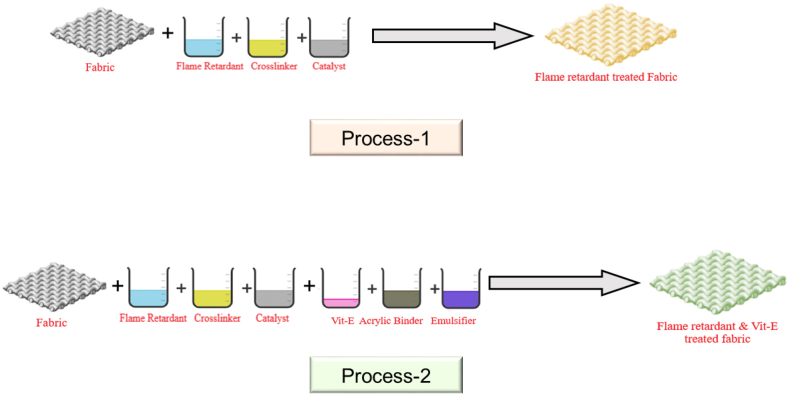
Overall processing steps of the flame retardant treatment with cotton and combination of flame retardant & Vitamin-E treatment preparation with cotton.

The fabrics were then subjected to double dipping and nipping in a pneumatic type heavy-duty vertical padding mangle (Rapid, China) at a pressure of 2 kg/cm^2^ and running speed of 6 m/min to achieve 80 % pick up. Following this, the padded fabrics were dried and cured in a laboratory mini dryer (Rapid, China) at 110 °C for 5 min and 160 °C for 1 min, respectively. To assess the durability of the finishes, half of the fabric samples were reserved for testing, while the rest were washed using American Association of Textile Chemists and Colorists (AATCC) standard reference detergent (9.7 gm/kg of water) for three cycles under normal washing machine conditions at 27 ± 3 °C and tumble dried according to AATCC-135. Before testing, all samples were preconditioned for 24 h at 20 ± 1 °C and 65 ± 2 % relative humidity. The overall processing steps of the flame retardant treatment with cotton and combination of flame retardant & Vitamin-E treatment preparation with cotton are shown in Fig. 2 through process-1 and process-2 respectively.

### 2.3 Characterizations

#### 2.3.1 Fourier-transform infrared (FTIR) spectroscopy

The cotton specimen's chemical composition was analyzed via a JASCO FT-IR 4700 spectrometer in attenuated total reflection (ATR) mode within the scanning range of 4000 to 500 cm^−1^. Additionally, a Hitachi SU 1510 scanning electron microscope (SEM) operating at 10 kV was employed to perform morphological studies on the cotton fabric specimens.

#### 2.3.2 Scanning electron microscope (SEM)

A surface morphological analysis was conducted on the control sample, as well as sample treated with flame retardant and a combination of flame retardant and vitamin E. This analysis was performed using a scanning electron microscope (FE-SEM) manufactured by HITACHI SU-1510 in Japan.

#### 2.3.3 Thermo-gravimetric analysis (TGA)

The SDT 650 equipment by TA instruments was used to conduct TGA under a nitrogen environment, with an approximate initial sample weight of 8 mg. The experiment was conducted by subjecting the sample to a continuous flow of nitrogen (N_2_) at a rate of 40 mL/min for balance and 60 mL/min for the sample, while heating it from 50 to 600 °C at a rate of 10 °C/min.

#### 2.3.4 Optical tensiometer

The “Attension Theta Lite” by Biolin Scientific, Sweden was used to conduct water contact angle assessment of control sample, flame retardant treated sample and combination of flame retardant & Vitamin-E treated sample.

#### 2.3.5 Fabric comfort properties test

In this study, various fabric comfort properties were evaluated using the fabric touch tester (FTT) method, including compression work, bending average rigidity, bending work, surface roughness amplitude, surface friction coefficient, compression recovery rate, compression average rigidity, surface roughness wavelength, and recovery average rigidity. The FTT apparatus, available from SDL Atlas allows for the measurement of fabric thickness, compression, bending, shearing, surface friction and roughness by a single test. Unlike other instruments, the FTT can evaluate multiple physical indices of the fabric sample with a single test. The FTT measurement method involves cutting the fabric sample in an L-shape and simultaneously measuring the indices in both the warp and weft directions. Details regarding the instrument's modules and index calculations are outlined in previous studies [47,48]. No standard FTT test protocol currently exists; therefore, the denim fabrics were tested following the equipment manufacturer's instructions [49]. The sample fabrics were stored for a minimum of 24 h under standard test conditions (20 ± 1 °C and RH 65 ± 5 %) and were cut and tested for each fabric sample option i. e flame retardant treated sample and combination of flame retardant & Vitamin-E treated sample, both before and after washing.

#### 2.3.6 Flammability test

The flammability assessment of the fabrics was carried out as per the guidelines of 16 CFR Part 1610 (Standard for the Flammability of Clothing Textiles). A 16 mm (5/8 in) flame was exposed to the specimen mounted at a 45° angle for a duration of 1 s. The burning of the specimen was allowed to continue until it burned its full length or until the stop thread was broken at a distance of 127 mm (5 in). The results of multiple specimens were averaged, and the flammability performance and surface characteristics of the sample were used to assign a class designation. A shorter time taken to burn indicates lower tendency for the fabric to burn and better flame retardant properties.

#### 2.3.7 Moisture management test

The moisture management performance of fabric samples was evaluated using the AATCC 195–2009 Standard Test Method with the “SDL-ATLAS Moisture Management Tester”. The control sample (raw fabric), flame retardant treated (before and after wash), and combined flame retardant and vitamin E treated (before and after wash) samples were conditioned at 25 ± 20 °C and 65 ± 2 % RH in accordance with ASTM D1776 standard practice for conditioning and testing textiles. The test solution was prepared by dissolving 9 gm of NaCl (USP grade) in 1 L of distilled water and adjusting the electrical conductivity to 16 ± 0.2 mS (ms) at 25 °C. The “measurement time” was set at 120 s for each sample. The moisture management properties of each fabric sample were evaluated by placing an 8 cm × 8 cm fabric sample between the two horizontal electronic sensors (top and bottom) of the “SDL-ATLAS Moisture Management Tester”. The test solution was dispensed in predetermined amounts and the parameters for the moisture management properties were recorded. These included wetting time (top and bottom surface), absorption rate (top and bottom surface), maximum wetted radius (top and bottom surface), spreading speed (top and bottom surface), and the accumulative one-way transport capability. The integrated software combined three measured performance characteristics to evaluate the moisture management properties of each sample. Each sample option i. e flame retardant treated sample and combination of flame retardant & Vitamin-E treated sample was tested five times and the means were recorded and plotted as a histogram.

#### 2.3.8 Fabric strength test

In this study, strength testing was conducted on the fabric samples using a tensile strength tester in accordance with ASTM D-1059, which is the standard test method for yarn number based on short-length specimens. Specimens were cut to dimensions of 100 ± 1 mm in width and 150 mm in length. Fabric samples were tested both before and after washing, and multiple samples were taken from two option (Flame retardant treated sample and combination of flame retardant & Vitamin E treated sample).

## 3 Results and discussion

### 3.1 Surface chemical analysis

**Fig. 3:**
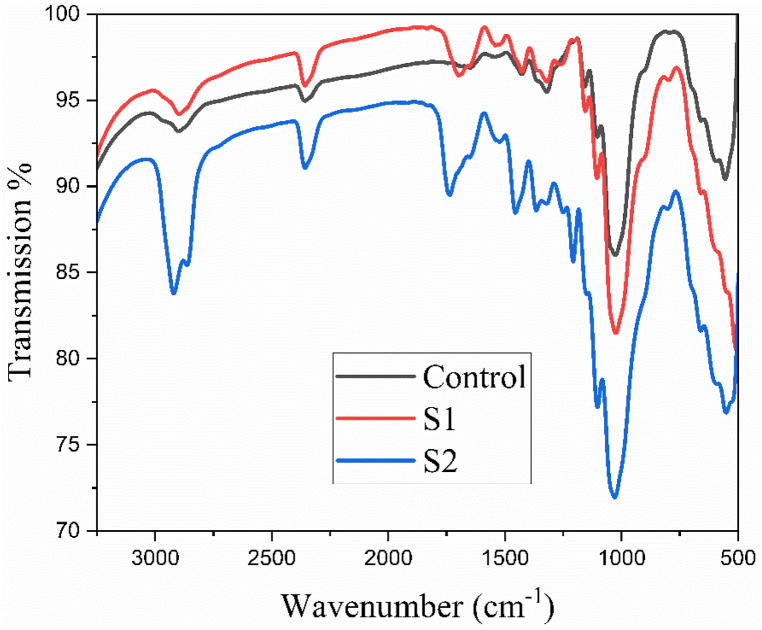
FTIR spectra of control, S1 (flame retardant), S2 (combine flame retardant & vitamin E treatment).

The surface chemical composition of the substrate was characterized using FTIR-ATR analysis. The FTIR spectra of control, S1 (flame retardant) and S2 (combine flame retardant & vitamin E treatment) are shown in Fig. 3. Fig. 3 shows the spectrum for the control cotton fabric with characteristic bands associated with the cellulose structure in the cotton fiber, including hydrogen bonded OH stretching at 3330 cm^−1^, asymmetric stretching of CH_2_ at 2900 cm^−1^, absorbed water molecules at around 1650 cm^−1^, CH wagging at 1320 cm^−1^, and CO stretching at 1026 cm-1 [50]. For the flame retardant treated fabrics, new characteristic peaks were observed at 1698 cm^−1^ for C=O stretching of amide present in the flame retardant agent [51]. Additionally, the spectra showed a distinct strong absorption band at 1540 cm^−1^ due to carbonyl stretching modes of carboxylate anion [19,52,53]. For the flame retardant and vitamin E treated fabrics, new characteristic bands appeared at 1455 cm^−1^ corresponding to skeletal vibration of phenyl ring system. Furthermore, two prominent bands were observed at 2920 cm^−1^ and 2863 cm^−1^, respectively, for the asymmetrical and symmetrical stretching mode of vibrations for aliphatic C–H bond such as CH_2_ and CH_3_ groups. Moreover, methyl symmetric bending was found at 1365 cm^−1^ [54].

### 3.2 Surface morphological analysis

**Fig. 4:**
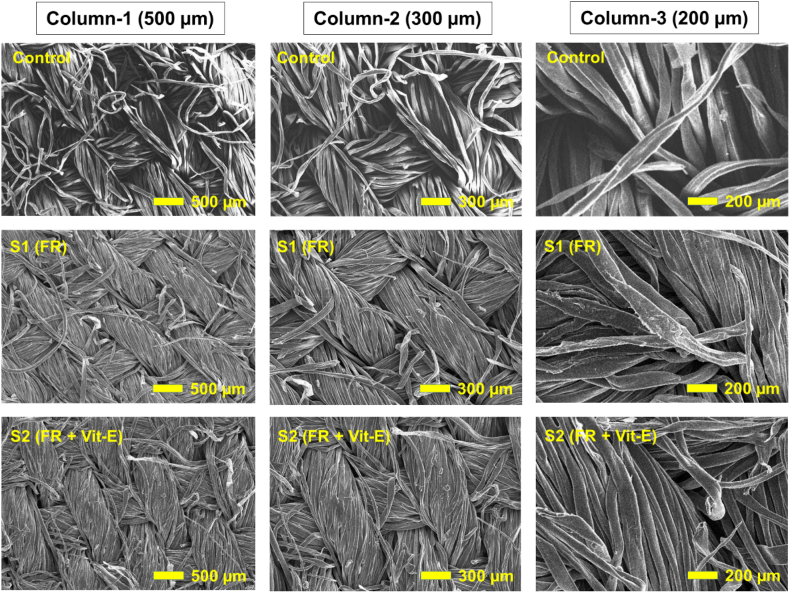
Scanning electron microscopic image of (a) control (b) S1(flame retardant) and (c) S2 (combine flame retardant & vitamin E treatment).

Scanning electron microscopy (SEM) was employed to examine the surface micro-morphologies of the control sample, S1 (flame retardant) sample, and S2 (flame retardant and vitamin E-treated) textiles and the resulting images are presented in Fig. 4. Column 1, 2, and 3 in Fig. 4 show SEM micrographs taken at 500 μm, 300 μm, and 200 μm magnifications, respectively for the control, flame retardant cotton (S1) and combination of vitamin E and flame retardant-treated (S2) samples. The images in Fig. 4 indicate that the control fibers have a rougher surface and are more loosely arranged than the flame retardant cotton (S1) and combination of vitamin E and flame retardant-treated (S2) samples [55].

In the first row of Fig. 4, the unmodified cotton surface exhibits slight rusting and fragmenting or aging, whereas the flame retardant cotton fiber appears more hydrated. The morphological changes observed in the second row for the flame retardant cotton sample are mainly attributed to the swelling of cotton fibers during the crosslinking reaction process. The surfaces of the cotton fibers in the FR cotton samples (in columns 1, 2, and 3) are smoother, as the fibers in FR cotton textiles are covered with a thin layer [55]. Furthermore, the addition of vitamin E in the third row of Fig. 4 makes the fabric appearance almost identical to that of the second row's flame retardant cotton sample. Essentially, in rows 2 and 3 for the S1 (flame retardant) and S2 (flame retardant and vitamin E-treated) textiles, a membrane structure exists between the fibers that connecting the textile fibers to each other. This suggests that vitamin E makes the fabric smoother without causing any significant changes in the fabric microstructure.

### 3.3 Thermogravemetric analysis

**Fig. 5:**
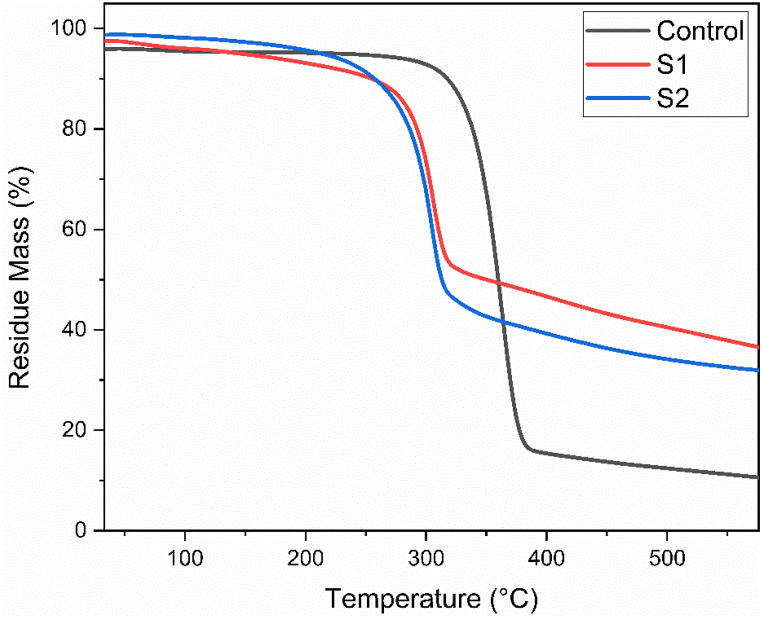
Thermogravemetric Analysis of control, S1 (flame retardant) and S2 (combine flame retardant & vitamin E treatment).

TGA (Thermogravimetric Analysis) is a thermal analysis technique used to study the thermal stability and decomposition behavior of materials. The TGA test involves measuring the weight changes of a material as it is heated in a controlled atmosphere. TGA curve of control, flame retardant treated (S1) and combination of flame retardant and Vitamin E (sample S2) treated sample are shown in Fig. 5. Here, Table 4 shows the decomposition temperature, temperature at highest rate of mass loss and char residue percentage at different temperature for control and treated sample.

**Table 4:** Decomposition temperature, temperature at highest rate of mass loss and char residue percentage at different temperature for control and treated sample.

Tempetature stages	Control	S1	S2
Intitial Decomposition Temperature (°C)	300	260	250
Temperature at highest rate of mass loss (T_max_) (°C)	381	320	317
Char residue at 476.5 °C	12.975 %	41.711 %	35.074 %
Char residue at 576 °C	10.587 %	36.537 %	31.923 %

Slight weight loss was observed at low temperature [56,57] suggested that the cellulose is predominantly damage at initial pyrolysis stage. The primary pyrolsis stage is observed for control fabric at 300 °C. During the second stage, the sample undergoes significant weight loss and pyrolysis of cellulose occurs within the crystalline area of the polymers. Glucose and several types of flammable gases are among the primary products generated during pyrolysis [51,57]. The highest rate of weight loss is observed at 381 °C for control fabric. When the temperature is increased, the material loses less mass over time because it loses water and carbon dioxide as well as produces carbonyl compound [56]. The TGA data indicates that the control sample underwent weight loss starting at 300 °C, with the maximum rate of weight loss occurring at 381 °C. By this point, approximately 75 % of the sample's mass had been lost. The weight loss increased to 87 % at 476 °C. The TGA data also indicates that the flame retardant sample (S1) underwent weight loss starting at 260 °C, with the maximum rate of weight loss occurring at 320 °C. By this point, approximately 47 % of the sample's mass had been lost. The weight loss increased to 87 % at 476.5 °C. On the contrary, combination of Flame retardant & vitamin E treated sample (S2) underwent weight loss starting at 250 °C, with the maximum rate of weight loss occurring at 317 °C. By this point, approximately 52 % of the sample's mass had been lost. At the stage of 576 °C, sample S2 lost 68 % of its mass where as both control & Sample S1 had lost 89 % and 63 % of its mass respectively. The reason of above phenomena might be described as the flame retardant treated cotton fabric samples displayed degradation at a lower temperature of 260 °C. The delay in the formation of volatile pyrolysis products during thermal degradation of the polymer is responsible for the variation in decomposition temperature observed between the control and sample S1 [57]. On the otherhand, the thermal decomposition temperature of sample S2 is lower than sample S1. If an object is more reachable by heat then it decreases the thermal decomposition temperature as well. Moreover, sample S1 and S2 displayed considerable char formation properties because at 576 °C sample S1 and S2 demonstrate 36.537 % and 31.923 % char residue respectively. Earlier studies indicated that better flame retardancy is linked to increased formation of char in this temperature range [58]. These TGA results demonstrate that sample S1 and S2 both contain considerable flame retardancy property.

### 3.4 Water contact angle

**Fig. 6:**
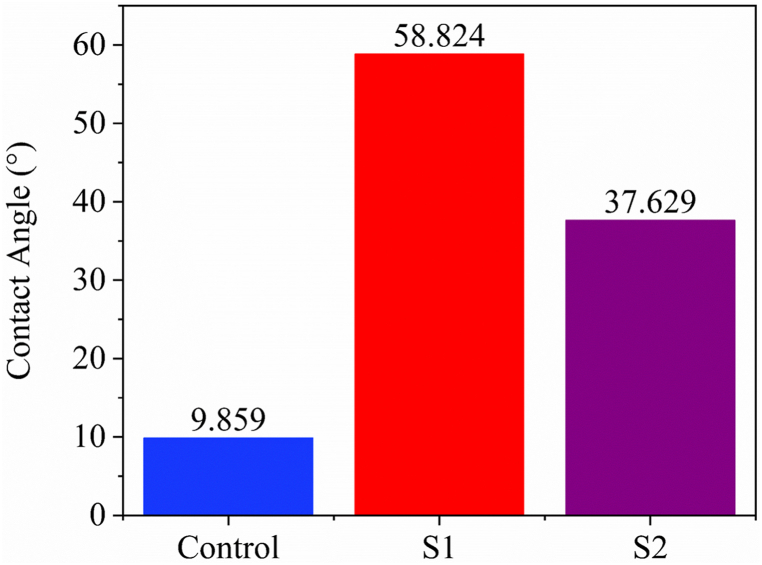
Water drop contact angle of the control, S1 (flame retardant) and S2 (combine flame retardant & vitamin E treatment).

Fig. 6. Illustrates the contact angle of water on the control, S1 (flame retardant) and S2 (combine flame retardant & vitamin E treatment) until it penetrates the fabric surfaces.

The water contact angle is a measure of the wettability of a material's surface. It determines whether a material is hydrophilic or hydrophobic. A hydrophilic material has a low contact angle, and water spreads quickly over its surface, indicating that it has high surface energy and an affinity for water. In contrast, a hydrophobic material has a high contact angle, and water beads up on its surface, indicating low surface energy and a repulsion of water. The value of contact angle for control, sample S1 and sample S2 are plotted at Fig. 6. It is observed that control sample experiences lowest contact angle that illustrate highest hydrophilicity of the material. This sample's rigorous pretreatment process during wet processing is the reason for its behavior, while the flame retardant sample (S1) exhibits a high contact angle that indicates its hydrophobic nature. The treatment used for flame retardancy involves a crosslinking agent that limits yarn mobility and causes yarns to stick together [38], which could hinder the fabric's ability to absorb water. Conversely, when vitamin E is added to the flame retardant treatment (S2), the contact angle decreases compared to the flame retardant sample. The reason for this decrease is the presence of alphatocopherol in vitamin E, which reduces the fabric's resistance to water intake.

### 3.5 Impact of vitamin E on fabric handle of flame retardant treated fabric

**Fig. 7:**
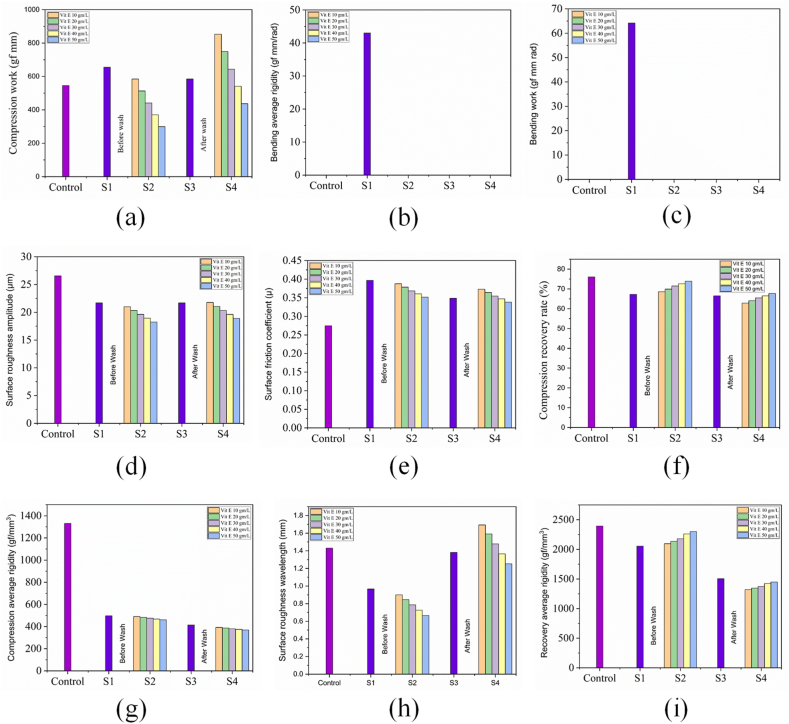
Fabric touch tester (FTT) results: (a) compression work of control; (b) bending average rigidity of control; (c) bending work of control; (d) surface roughness amplitude; (e) surface friction coefficient; (f) compression recovery rate; (g) compression average rigidity; (h) surface roughness wavelength; (i) recovery average rigidity of control, flame retardant (before and after wash), combine flame retardant & vitamin E treatment (before and after wash).

The fabric touch tester (FTT) results (compression work of control, bending average rigidity of control, bending work of control, surface roughness amplitude, surface friction coefficient, compression recovery rate, compression average rigidity, surface roughness wavelength, recovery average rigidity) of control, flame retardant (before and after wash) and combine flame retardant & vitamin E treatment (before and after wash) are shown in Fig. 7(a), 7(b), 7(c), 7(d), 7(e), 7(f), 7(g), 7(h) and 7(i) respectively. S1, S2, S3 and S4 represents Flame retardant finish sample, combine flame retardant & vitamin E treated sample, Flame retardant finish sample (after wash) and combine flame retardant & vitamin E treated sample (after wash) respectively.

Fabric compression denotes the change in fabric thickness under altered loads and delivers an illustration of fabric softness or fabric fullness. So compression work can measure the softness of the fabric as well. Lower value of compression work refers The value of compression work for sample (control, S1, S2, S3 and S4) are plotted as shown in Fig. 7(a). The lower value of compression work refers less work need to be performed to compress the fabric and vice-versa. The current investigation revealed that sample S1 shows highest compression work among all sample because it becomes hard once flame retardant is treated onto the fabric. The reason behind the hardness of sample S1 is the using of crosslinking agent during flame retardant treatment [50]. Again sample S2, with vitamin E treatment of 50 g/L showed lowest value of compression work among all sample because fabric becomes softer once vitamin E was added into the fabric along with flame retardant. The reason behind the softness of sample S2 might be the presence of alpha tocopherol in the fabric which comes from Vitamin-E. This alpha tocopherol might be effectively softening the fabric which showed the low value of compression work for sample S2. Furthermore, sample S3 and sample S4 showed higher value after wash because once vitamin E is exerted then more compression work was needed to change the fabric thickness.

Bending average rigidity defines the flexibility of fabric. Fabric having higher bending rigidity considered as stiff fabric and vice versa [50]. The bending average rigidity values for the control and different treated samples (S1, S2, S3, S4) have been plotted and presented in Fig. 7(b). The lower value of bending rigidity refers less force is needed to bend the fabric and vice versa. The experimental results indicate that the bending average rigidity of sample S1 is higher than that of the other samples. This might be happening due to formation of bridges during the crosslinking process between the fibres which makes hard to bend the fabric [38]. Sample S2 of any concentration of Vitamin E treatment exhibited no bending average rigidity. The reason of getting no bending rigidity for sample S2 might be the presence of alpha tocopherol in the fabric which comes from Vitamin-E. No bending average rigidity was also found for sample S3, S4. The reason of this phenomenon might be washing effect. Once washing was performed then bridge between fibre is loosened which results no bending rigidity.

Bending work refers to the amount of work required to bend the specimen. Fabric having higher bending work considered as stiff fabric and vice versa [50]. The value of bending work for sample (control, S1, S2, S3 and S4) are plotted as shown in Fig. 7(c) The lower value of bending work refers less work is needed to bend the fabric and vice versa. The experimental results indicate that the bending average rigidity of sample S1 is higher than that of the other samples. This might be happening due to formation of bridges during the crosslinking process between the fibres which makes hard to bend the fabric [38]. Sample S2 of any concentration of Vitamin E treatment exhibited no bending work. The reason of getting no bending work for sample S2 might be the presence of alpha tocopherol in the fabric which comes from Vitamin-E. No bending work was also found for sample S3, S4. The reason of this phenomenon might be washing effect. Once washing was performed then bridge between fibre is loosened which results no bending work.

Surface roughness amplitude defines the maximum extent of a fibre wave measured from the flat surface of fabric. Fabric having higher surface roughness amplitude considered as stiff fabric and vice versa. The surface roughness amplitude for each sample (control, S1, S2, S3, S4) is graphically presented in Fig. 7(d). The lower value of surface roughness amplitude refers minor extent of irregular fibre wave from the flat surface of fabric and the higher value of surface roughness amplitude refers major extent of irregular fibre wave. From the present study it was observed that control sample shows highest surface roughness amplitude among all others sample because of presence maximum intra-fibre distance. For sample S1, fibre to fibre bridges are formed by crosslinking process which might be reduced the intra-fibre distance in a fibre wave. Sample S2, with Vitamin E treatment of 50 g/L exhibited lowest surface roughness amplitude than sample S1. The reason of lowering the surface roughness amplitude of sample S2 might be the presence of alpha tocopherol in the fabric which comes from Vitamin-E. Vitamin E might reduce the intra fibre distance more efficiently which results the lower surface roughness amplitude than flame retardant treated sample S1. Furthermore, for sample S3 and S4 surface roughness amplitude was increased again. Once washing is performed on sample S3 and S4 then flame retardant and vitamin E were exerted from the sample. As a result, intra fibre distance increases which results higher surface roughness amplitude in comparing with sample S1 and S2.

Surface friction coefficient is a ratio used to quantify the frictional force in relation to the normal force [38,50,59,60]. Fabric having higher surface friction coefficient considered as stiff fabric and vice versa. The value of surface friction coefficient for sample (control, S1, S2, S3, S4) are plotted as shown in Fig. 7(e). The lower value of surface friction coefficient refers lower frictional force and vice versa. From the present study it was observed that control sample has lowest coefficient of friction among all other sample. For sample S1, surface friction coefficient was increased because when flame retardant chemical was added then fibre to fibre bridges are formed by crosslinking process that results a harsh fabric. Sample S2, with Vitamin E treatment of 50 g/L exhibited lowest surface friction coefficient than sample S1. The reason behind the lowering of coefficient of friction for sample S2 might be the presence of alpha tocopherol in the fabric which comes from Vitamin-E. Vitamin E might reduce the frictional force in the fabric sample. Furthermore, for sample S3 and S4 surface coefficient of friction was decreased again. Once washing is performed on sample S3 and S4 the chemical is exerted from the sample. As a result, frictional force decreases which results lower coefficient of friction in comparing with sample S1 and S2.

The percentage of thickness change on removal of the load relative to the change in thickness between the preload and the main load defines the compression recovery rate. The higher value of compression recovery rate refers maximum energy is stored on the fibre core due to compression and vice versa. Fabric having higher compression recovery rate considered as soft fabric and vice versa. The value of compression recovery rate for sample (control, S1, S2, S3, S4) are plotted as shown in Fig. 7(f). From the present study it was observed that control sample shows highest compression recovery rate among all others sample because of storage maximum energy into the fibre core due to compression. For sample S1, compression recover rate is lessened. This phenomenon might be occurred due to storage of less amount energy because of adding crosslinking agent that has an impact of adhering of yarn with one another by reducing the yarn mobility [38]. As a result, yarn of sample S1 has become unable to show recovery tendency after compression. Furthermore, for sample S2, with Vitamin E treatment of 50 g/L exhibited comparatively highest compression recovery rate than sample S1 because alpha tocopherol from vitamin E might increase the yarn mobility. Again vitamin E might reduce the crystalline region of cellulose more efficiently which results higher magnitude of compression recovery rate. As a result, yarn of sample S2 has become more enable to show recovery tendency after compression. Furthermore, according to the Newton's third law of motion, sample S2 experiences maximum deformation in terms of thickness due to softness that's why it exerted maximum compression recovery rate. Furthermore, for sample S3 and S4, compression recovery rate was decreased again. Once washing is performed on sample S3 and S4 then flame retardant and vitamin E were exerted from the sample. As a result, intra fibre distance increases which results lower compression recovery rate than the unwashed flame retardant treated sample S1 and flame retardant along with vitamin E treated sample S2.

Compression average rigidity discusses about the forces needed to compress per millimeter of fabric sample. Fabric having lower compression average rigidity considered as soft fabric and vice versa. The lower the value of compression average rigidity, the lower the force required to compress the fabric sample, and vice versa. The value of compression average rigidity for sample (control, S1, S2, S3, S4) are plotted as shown in Fig. 7(g). From the present study it was observed that control sample shows highest compression average rigidity among all other sample because of the requirement of maximum forces to compress the control sample. For sample S1, compression average rigidity is lessened. This phenomenon can be occurred due to requirement of less amount of forces to compress the sample S1. Furthermore, for sample S2, with Vitamin E treatment of 50 g/L exhibited lowest compression average rigidity in compare with sample S1. This phenomenon clearly defines that sample S2 is comparatively softer than sample S1 because less force is required to compress the fabric sample S2 in compare with sample S1. The reason behind this phenomenon might be the presence of alpha tocopherol in the fabric which comes from Vitamin-E that ensures the low force requirement. Besides soft fabric must have low crystalline region as well as high amorphous region. On the other hand stiff fabric must contain high crystalline region as well as low amorphous region [61]. Vitamin E might reduce the crystalline region of cellulose more efficiently which results lower compression average rigidity that creates a soft fabric. Furthermore, for sample S3 and S4, compression average rigidity was decreased again. Once washing is performed on sample S3 and S4 then flame retardant and vitamin E were exerted from the sample and orientation of fibre might drastically change which results the reduction of crystalline region of cellulose in sample fabric. This structural change of cellulose might result the lower compression average rigidity.

The surface roughness wavelength can be defined as the distance between two consecutive crests on the surface of a fabric. Fabric having lower surface roughness wavelength might be considered as smooth fabric and vice versa. The value of surface roughness wavelength for sample (control, S1, S2, S3, S4) are plotted as shown in Fig. 7(h) A lower value of surface roughness wavelength indicates that the distance between one wave crest and the next is shorter, while a higher value indicates a longer distance between wave crests. Wavelength of lower distance create more number of wave in a specific unit area that might results smooth touch of sample fabric and wavelength of higher distance create less number of wave in a specific unit area which might results unpleasant feeling of sample fabric. From the present study it was observed that control sample shows higher surface roughness wavelength in comparison with other sample except sample S4. In comparison with sample S1 and S2, Sample S2 experiences lower wave length that indicates smoother surface of fabric. The reason behind this phenomenon might be the presence of alpha tocopherol in the fabric which comes from Vitamin-E. Vitamin E might reduce the wavelength more efficiently which results the lower surface roughness wavelength in comparing with sample S1. Furthermore, for sample S3 and S4 surface roughness wavelength was increased again. Once washing is performed on sample S3 and S4 then flame retardant and vitamin E were exerted from the sample which results higher surface roughness wavelength. As a result, S3 and S4 sample experiences harsh feeling during touch.

Recovery average rigidity discusses about the forces reflected during recovery of fabric sample per millimeter. Fabric having higher recovery average rigidity considered as soft fabric and vice versa. Besides soft fabric must have low crystalline region as well as high amorphous region. On the other hand stiff fabric must contain high crystalline region as well as low amorphous region [61]. The higher value of recovery average rigidity refers maximum forces is reflected from the fabric sample and vice versa. The value of recovery average rigidity for sample (control, S1, S2, S3, S4) are plotted as shown in Fig. 7(i). From the present study it was observed that control sample shows highest recovery average rigidity among all other sample because of the reflection of maximum forces from the control sample. For sample S1, compression average rigidity is lessened. This phenomenon can be occurred due to reflection of less amount of forces to compress the sample S1. Furthermore, for sample S2, with Vitamin E treatment of 50 g/L exhibited higher recovery average rigidity in compare with sample S1. This phenomenon clearly defines that sample S2 is comparatively softer than sample S1 because more force is reflected from the fabric sample S2 in compare with sample S1. The reason behind this phenomenon might be the presence of alpha tocopherol in the fabric which comes from Vitamin-E that ensures the higher reflection of force. Vitamin E might reduce the crystalline region of cellulose more efficiently which results higher recovery average rigidity. Furthermore, for sample S3 and S4, recovery average rigidity was decreased again. Once washing is performed on sample S3 and S4 then flame retardant and vitamin E were exerted from the sample and orientation of fibre might drastically change which results again rising of crystalline portion of cellulose in sample fabric. This structural change of cellulose might result the lower recovery average rigidity. Due to its superior softness compared to other concentrations, 50 g/L vitamin E is utilized for conducting flame retardancy analysis (FRA), moisture management test (MMT), and fabric strength test denoted as S2, S4 for before wash and after wash respectively.

### 3.6 Flame retardancy analysis

**Fig. 8:**
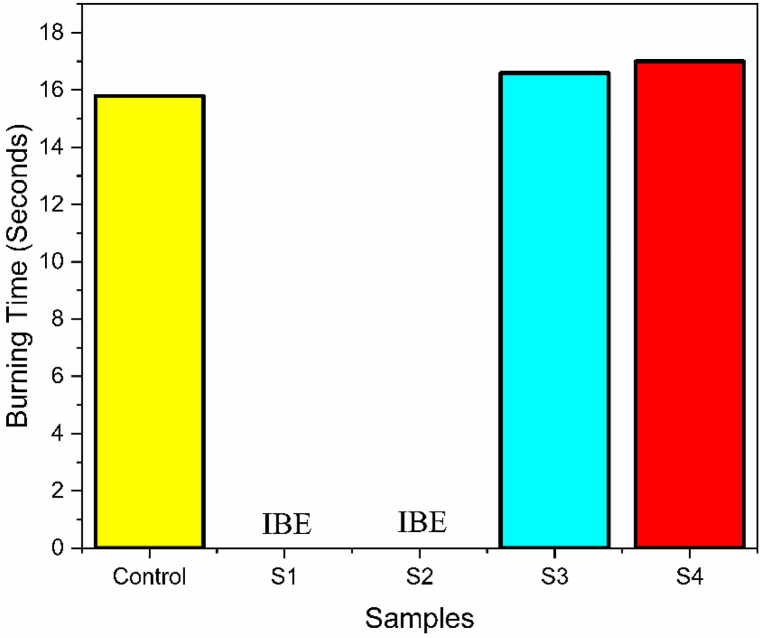
Flammability test results: Burn time of control, flame retardant (before and after wash), combine flame retardant & vitamin E treatment (before and after wash) at (a) course wise (b) wales wise.

**Fig. 9:**
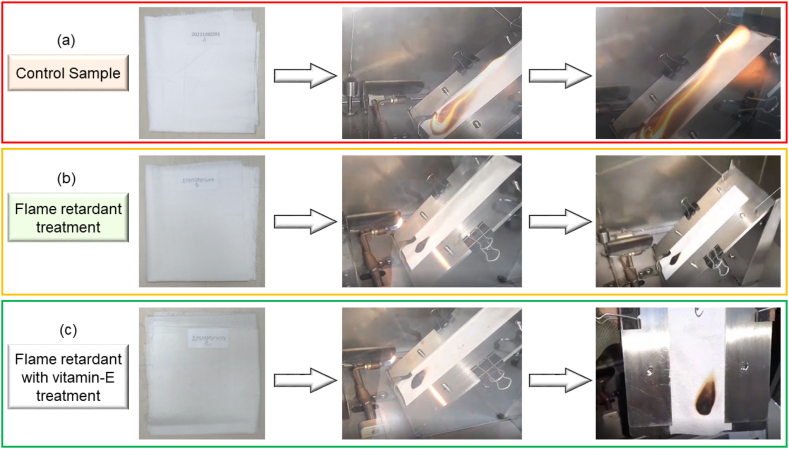
Flammability or burning test of the control, flame retardant and combine flame retardant with vitamin E treatment of the cotton samples.

Flammability is a characteristic or property of a substance that describes its susceptibility to ignite and sustain combustion when exposed to an external heat source, an open flame, or other sources of ignition. Here S1, S2, S3 and S4 represents Flame retardant finish sample, combine flame retardant & vitamin E treated sample, Flame retardant finish sample (after wash) and combine flame retardant & vitamin E treated sample (after wash) respectively. The value of flame retardancy for sample (control, S1, S2, S3, S4) are plotted as shown in Fig. 8. The 15.8 s burn time observed for control is consistent with materials lacking flame retardant treatment. The absence of fire-resistant additives or treatments in this sample contributed to its limited fire resistance. Materials like control sample, without any fire-retardant properties, are more susceptible to combustion and less capable of self-extinguishing. The “IBE” i. e “ignited but extinguished” result obtained for sample S1 is noteworthy considering its flame-retardant treatment. In the case of sample S1, the presence of a flame retardant likely contributed to its self-extinguishing behavior. Furthermore, Sample S2, treated with both a flame retardant and vitamin E, also exhibited the “IBE” result. While vitamin E is not typically associated with flame-retardant properties, its inclusion may have secondary effects on fire resistance. Vitamin E is known for its antioxidant properties, which can potentially mitigate oxidative reactions during combustion. The combination of a flame retardant and vitamin E in sample S2 may have synergistic effects, contributing to its self-extinguishing behavior. The reason of above phenomena at sample S1 and S2 must be the presence of flame retardant chemical which generates carbonaceous compound (solid char) after the combustion process. This carbonized layer quarantined sample fabrics to get burn and protect the cellulose polymer from flame attack [50]. The treatment of Vitamin E along with flame retardant doesn't hamper the flame retardancy property of the used flame retardant chemical. This impression mentions the well-being of using Vitamin E with flame retardant treated fabric. Furthermore, sample S3 and S4 shows burn time more than 3.5 s which also establish the efficacy of flame retardant and combine flame retardant and vitamin E treated fabric even after 3 cycle wash. The flammability or burning test of the control, flame retardant and combine flame retardant with vitamin E treatment of the cotton samples are shown in Fig. 9 (a), 9 (b) and 9(c) respectively.

### 3.7 Impact of vitamin E on moisture management of flame retardant treated fabric

**Fig. 10:**
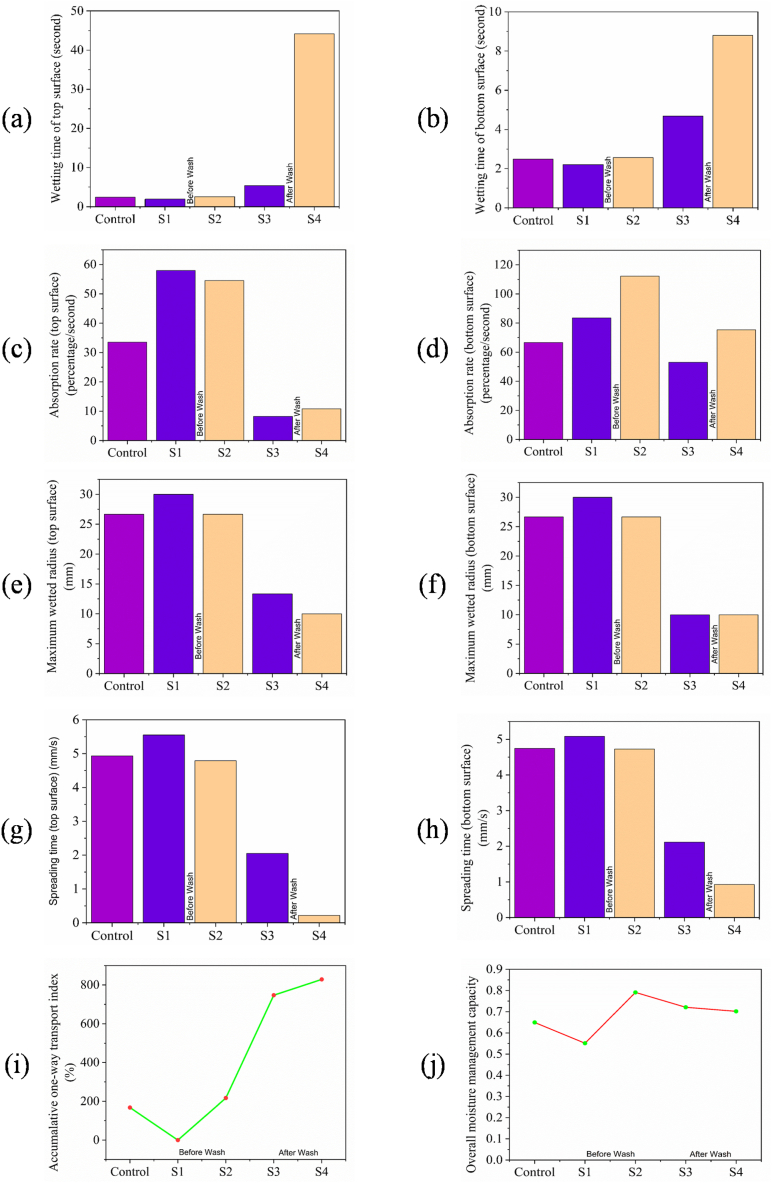
Moisture management test results (a) wetting time (top surface); (b) wetting time (bottom surface); (c) absorption rate (top surface); (d) absorption rate (bottom surface); (e) maximum wetted radius (top surface); (f) maximum wetted radius (bottom surface); (g) spreading time (top surface); (h) spreading time (bottom surface); (i) accumulative one way transport index (AOTI); (j) overall moisture management capacity (OMMC) of control, flame retardant (before and after wash), combine flame retardant & vitamin E treatment (before and after wash); Grading: ≥120 = No wetting; 20–119 = Slow; 5–19 = Medium; 3–5 = Fast; <3 = Very Fast [[62], [63], [64]].

The moisture management test results (wetting time on the top surface, wetting time on the bottom surface, absorption rate at the top surface, absorption rate on the bottom surface, maximum wetted radius on the top surface, maximum wetted radius at the bottom surface, spreading time at the top surface, spreading time at the bottom surface, accumulative one-way transport index and overall moisture management capacity) of control, flame retardant (before and after wash), combine flame retardant & vitamin E treatment (before and after wash) are illustrated in Fig. 10(a), 10(b), 10(c), 10(d), 10(e), 10(f), 10(g), 10(h), 10(i) and 10(j) respectively. S1, S2, S3 and S4 represents flame retardant finish sample, combine flame retardant & vitamin E treated sample, flame retardant finish sample (after wash) and combine flame retardant & vitamin E treated sample (after wash) respectively. The results obtained from the present experimental procedure are contingent on the water resistance, water repellency, and water absorption properties of the fabric structure, including its geometry, internal arrangement, as well as fiber and thread wicking characteristics. This study presents a pictorial representation of the moisture management attributes of untreated samples, those treated with flame retardant, and those treated with a combination of vitamin E and flame retardant. The upper surface parameter pertains to the part of the fabric that comes into direct contact with the skin during use of the product or garment, whereas the lower surface indicates the surface that is predominantly exposed when the product or garment is in use.

The wetting time of fibrous porous materials is defined as the duration required to wet both the top and bottom surfaces [65]. Fabrics that exhibit faster wetting times are classified as “very fast” water-consumable fabrics, while those with slower wetting times are classified accordingly. Surface tension and contact angle are the factors that determine wetting time. The value of wetting time for sample (control, S1, S2, S3, S4) are plotted as shown in Fig. 10(a) and (b). Control sample, Sample S1 and S2 experiences “very fast” wetting time as their wetting time lies in same range both for top and bottom surface. This indicates that combination of vitamin E with flame retardant doesn't deteriorate the wetting time. From the present study it was observed that control sample, sample S1 and sample S2 optimized by 50 g/L vitamin E, both experiences “very fast” wetting time in compare to sample S3 and S4. This phenomenon could be occurring because control sample, sample S1 and S2 experience a lower degree of cohesion force compared to adhesion force to the surface of the fabric. Furthermore, control sample, sample S1 and S2 might offer lower contact angle i.e. 9.859°, 58.824° and 37.629° respectively which results lower wetting time [66]. Furthermore, the high attraction between the liquid and the fabric surface is responsible for faster wetting and is referred to as low fiber surface energy fabric. Due to “very fast” wetting, combine treatment of Vitamin E and Flame retardant sample can be classified as low fibre surface energy bearing fabric. The treatment of vitamin E is hypothesized to reduce the surface energy of the fibers [67].

The absorption rate is defined as the weight of water absorbed relative to the weight of the dry material. Fabric having higher wetting time recognize as very fast water consumable fabric and vice versa. Cellulosic fibres experiences swelling due to water absorption shows reduction in stiffness of cellulosic fibres [68]. It implies that the more absorption rate of sample fabric results more soft fabric and vice versa. The absorption rate values for the control, S1, S2, S3, and S4 samples are depicted in Fig. 10(c) and (d). The current investigation reveals that control sample, sample S1 and sample S2 optimized by 50 gm/L vitamin E experiences “fast” absorption rate as they have low contact angle (<90°) i.e. 9.859° 58.824° and 37.629° respectively. On the other hand, bottom surface of sample S2 experiences higher absorption rate among all samples. This indicates that combination of vitamin E with flame retardant doesn't deteriorate the liquid absorption rate. Furthermore, absorption rate at bottom surface shows highest value for sample S2 that defines “very fast” water absorption rate which expresses maximum softness of fabric sample treated by vitamin E. The observed phenomenon may be attributed to a modification in the cover factor resulting from the application of vitamin E, which leads to increased porosity of the fabric and, in turn, an elevation in the absorption rate [67].

The maximum wetted radius is a critical factor that influences the drying rate of fabric. Fabrics with larger wetted radii are known to dry more quickly than those with smaller radii. Faster drying rates correspond to the efficient transport of liquid from the inner surface to the outer surface, resulting in minimal liquid retention on the inner surface and easy transfer of liquid moisture from the outer fabric surface into the environment. As a result, fabrics with larger wetted radii tend to dry more rapidly [67]. The value of maximum wetted radius for sample (control, S1, S2, S3, S4) are plotted as shown in Fig. 10(e) and (f). Sample S1 and sample S2 optimized by 50 g/L vitamin E experiences “very fast wetting” as their maximum wetted radius value lies in same range both for top and bottom surface. The current investigation reveals that control sample, sample S1 and sample S2 optimized by 50 gm/L vitamin E experiences “very fast wetting” as they have low contact angle (<90°) i.e. 9.859° 58.824° and 37.629° respectively. This indicates that combination of vitamin E with flame retardant doesn't deteriorate the maximum wetted radius of fabric sample. This phenomenon might be happening as there is no surface distortion is performed due to vitamin E application. Moreover, sample S3 and S4 shows lower wetted radius because fabric surface is distorted due to washing operation.

Spreading time illustrates the sweaty environment of fabric. Higher spreading time indicates quick drying of fabric as moist are not stationary in the fabric top surface. As a result fabric provides better feeling to the wearer [67]. The value of spreading time for sample (control, S1, S2, S3, S4) are plotted as shown in Fig. 10(g) and (h). Sample S1 and sample S2 optimized by 50 g/L vitamin E experience “very fast” spreading as their value of spreading time lies in same range both for top and bottom surface. The current investigation reveals that control sample, sample S1 and sample S2 optimized by 50 gm/L vitamin E experiences “very fast” spreading as they have low contact angle (<90°) i.e. 9.859° 58.824° and 37.629° respectively. This indicates that combination of vitamin E with flame retardant doesn't deteriorate the spreading time of fabric sample. This phenomenon might be happening as there is no surface distortion is performed due to vitamin E application. Moreover sample S3 and S4 shows lower spreading time because fabric surface is distorted due to washing operation [67].

The accumulative one-way transport index (AOTI) provides a direct indication of the fabric's liquid transmission ability from the interior to the exterior layer [69]. Fabrics with high AOTI values can effectively manage perspiration by allowing it to pass through the fabric from the inner material side to the outer side. This keeps the wearer's skin dry by removing perspiration from the skin's surface. Positive and high AOTI values indicate that liquid sweat can be easily and quickly transported from human skin to the outer surface. The AOTI values for the control, S1, S2, S3, and S4 samples are presented in Fig. 10(i). All fabrics, except for S1, exhibit “very good” to excellent ratings in AOTI. S1, however, has a poor rating and a negative AOTI value, as shown in Fig. 10(i). The cause of this phenomenon may be attributed to the alteration of fabric thickness resulting from the application of flame retardant treatment and the combined treatment of flame retardant and vitamin E [70].

The Overall Moisture Management Capacity (OMMC) refers to a fabric's comprehensive capacity to regulate the transport of liquid moisture. Fabrics with higher OMMC values can efficiently manage liquid perspiration accumulation and provide a dry sensation to the wearer. This feature is essential for athletes because sweat production is ongoing but extreme due to body movement and exertion [67]. The value of Overall Moisture Management Capacity (OMMC) for sample (control, S1, S2, S3, S4) are plotted as shown in Fig. 10(j). It was observed that sample S2 optimized by 50 gm/L vitamin E experiences highest OMMC value among all other sample that clearly defines that combine treatment of fire retardant and Vitamin E enhances the fabric's moisture absorption capacity from the skin, facilitates the transportation of moisture to the outer surface, and facilitates its release into the surrounding environment. The reason of this phenomena is the combine result of “very fast” wetting time, “fast” absorption rate, “very fast wetting” for maximum wetted radius, “very fast” spreading time, “very good” rating for accumulative one-way transport index (AOTI).

### 3.8 Fabric strength

**Fig. 11:**
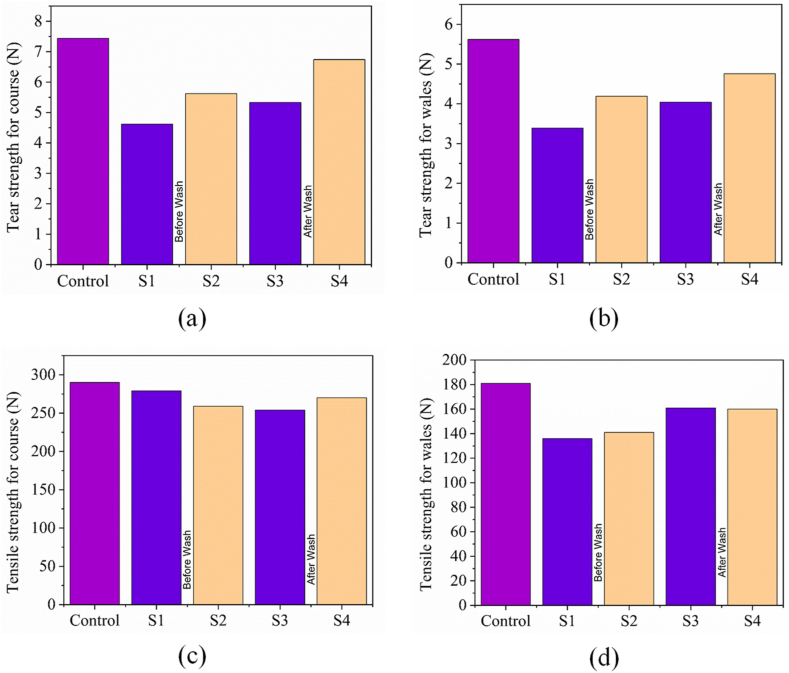
Tear strength of (a) course wise; (b) wales wise and tensile strength of (c) course wise; (d) wales wise of control, flame retardant (before and after wash), combine flame retardant & vitamin E treatment (before and after wash).

The tear strength (course wise and wales wise) and tensile strength (course wise and wales wise) of control, flame retardant (before and after wash), combine flame retardant & vitamin E treatment (before and after wash) are shown in Fig. 11(a) and (b), 11(c) and (d), respectively. S1, S2, S3 and S4 represents Flame retardant finish sample, combine flame retardant & vitamin E treated sample, Flame retardant finish sample (after wash) and combine flame retardant & vitamin E treated sample (after wash) respectively. The tearing resistance of a fabric in the weft or warp direction under specific conditions is referred to as tear strength, according to Ref. [71]. In order to assess the strength and elasticity of a fabric, tensile strength testing is carried out by measuring the force required to elongate and break a sample of the fabric [72]. Higher the tear strength expresses more strength occupied fabric and vice-versa. The value of tear strength in course wise and wales wise is plotted at Fig. 11(a) and (b) for sample (control, S1, S2, S3, S4). It was observed that sample S2 optimized by 50 gm/L vitamin E and sample S4 experiences higher tear strength for both course and wales direction of fabric even after 3 cycle wash in comparing with corresponding sample S1 and sample S3. The reason of significantly lowering the tearing strength for flame retardant treated sample is the acidic effect of padding bath which results acid hydrolysis of cellulose due to breakdown of cellulose chain. High-temperature curing at 160 °C can lead to the brittleness of cotton, as reported by Ref. [38]. The tearing strength of fabrics decreases with increasing curing temperature, according to Ref.[51]. Moreover, the flame retardant treatment of fabrics involves a crosslinking agent that reduces yarn mobility and causes yarns to stick together, which may decrease tearing strength [50]. In contrast, the presence of alpha-tocopherol from vitamin E may enhance the smoothness of fibers, reduce inter-fiber friction, and improve yarn mobility, which could explain why sample S2 showed higher tearing strength.

The value of tensile strength is shown at Fig. 11(c) and (d) for sample (control, S1, S2, S3, S4). Higher the tensile strength expresses more strength is needed to extend the fabric and vice versa. Compared to the control fabric, the flame retardant and combination of flame retardant & vitamin E treated fabrics exhibited significantly lower tensile strength. The decrease in tensile strength can be attributed to the use of a crosslinking agent in the flame retardant treatment. This agent is prepared using melamine resin and formaldehyde, which can weaken the strength of the cotton fibers by damaging the cellulose polymer chain [[73], [74], [75]]. Moreover, the high curing temperature may cause the lower tensile strength of sample S1 and sample S2.

## 4 Conclusions

Softness is a desirable characteristic of textile fabrics that can be achieved through the use of softeners. However, the use of conventional softeners may negatively affect the fabric strength and moisture management properties of the fabric. This study explores the use of Vitamin E as a natural softener for cotton fabrics, which is not only improves the fabric's softness but also enhances its moisture management properties without compromising the fabric's flammability as well as fabric strength. The results of the study show that Vitamin E treatment significantly improves the fabric's softness and moisture management properties while it might provide potential health benefits to the wearer due to Vitamin E's skin permeability. This finding suggests that Vitamin E could be a promising alternative to traditional softeners in textile finishing, offering a more natural and beneficial approach to achieving softness in fabrics.

## Data availability statement

The raw/processed data required to reproduce these findings can be shared at any time in order to helps other researchers.

## CRediT authorship contribution statement

**A.T.M. Gulam Moula:** Conceptualization, Data curation, Formal analysis, Investigation, Methodology, Resources, Writing – original draft, Writing – review & editing, Visualization. **Md. Abdullah Al Mamun:** Conceptualization, Formal analysis, Project administration, Supervision, Visualization, Writing – review & editing. **Md. Humayun Kabir Khan:** Writing – review & editing. **Md. Dulal Hosen:** Conceptualization, Data curation, Formal analysis, Investigation, Methodology, Resources, Software, Visualization, Writing – original draft, Writing – review & editing. **Md. Abu Bakar Siddiquee:** Conceptualization, Formal analysis, Project administration, Supervision, Visualization.

## Declaration of competing interest

The authors declare that they have no known competing financial interests or personal relationships that could have appeared to influence the work reported in this paper.
